# The effect of the association between CETP variant type and alcohol consumption on cholesterol level differs according to the ALDH2 variant type

**DOI:** 10.1038/s41598-022-19171-y

**Published:** 2022-09-06

**Authors:** Min-Gyu Yoo, Ji Ho Yun, Soo Kyung Koo, Hye-Ja Lee

**Affiliations:** Division of Endocrine and Kidney Disease Research, Department of Chronic Disease Convergence Research, Korea National Institute of Health, Korea Disease Control and Prevention Agency, 187 Osongsaengmyeong 2-ro, Osong-eup, Cheongju-si, Chungcheongbuk-do 28159 Republic of Korea

**Keywords:** Genetic variation, Risk factors, Genetic association study, Genotype

## Abstract

Alcohol consumption is associated with a high increased lipid profile and this association may depend on genetic risk factors. In this study, we aimed to assess the effects of genetic variation associated with alcohol consumption on lipid profiles using data from two Korean population studies. We performed a genotype association study using the HEXA (*n* = 51,349) and KNHANES (*n* = 9158) data. Genotype analyses of the two sets of Korean population data showed associations of increased total cholesterol and high-density lipoprotein (HDL)-cholesterol with *CETP* rs708272. The HEXA and KNHANES populations revealed differences in HDL cholesterol according to the presence of *CETP* rs708272, independent of *ALDH2* rs671 and alcohol consumption. In contrast, total cholesterol levels were associated with alcohol consumption and *ALDH2* rs671 in men with *CETP* rs708272 (CT and TT genotypes). Furthermore, in drinkers with *ALDH2* rs671 (GA and AA genotypes), higher total cholesterol was associated with the *CETP* rs708272 TT minor homozygous genotype based on both HEXA and KNHANES data. Our findings demonstrated that alcohol consumption and genetic variation in either *CETP* or *ALDH2* may be associated with cholesterol levels. We hope these findings will provide a better understanding of the relationship between alcohol consumption and cholesterol according to each individual’s genetic background.

## Introduction

Alcohol consumption is a major environmental factor that is associated with health problems. Greater alcohol consumption exacerbates the lipid profile^1^, which has been linked to various diseases, including dyslipidemia and cardiovascular diseases^2,3^. Previous studies have reported that alcohol consumption is associated with improved high-density lipoprotein cholesterol (HDL-C) levels, but also with increased triglyceride (TGs)^4^ and low-density lipoprotein (LDL)^5^. The association between alcohol consumption and total cholesterol remains controversial, with several studies either showing no effect^6^ or increased cholesterol levels. Thus, it is necessary to clarify the association between alcohol consumption and lipid profiles and the related mechanisms in large groups representing various races/ethnicities.

Cholesterol levels are associated with both genetic and environmental factors. Among them, cholesteryl ester transfer protein (CETP; also called plasma lipid transfer protein), which circulates in plasma, is mainly bound to HDL-C and facilitates the transfer of cholesteryl ester particles to apolipoprotein B (including LDL and very low-density lipoprotein (V-LDL)) in exchange for TGs^7^. In general, changes in the activity and concentration of CETP decrease the plasma HDL-C levels and increase the plasma LDL-cholesterol (LDL-C) levels. Additionally, genetic variations are related to its regulation^8^. *CETP* rs708272 (also called *Taq1B*) is a C-to-T substitution at the 279th nucleotide of intron 1 and is the most thoroughly studied variant of this gene. A previous study identified an association between increased HDL-C levels or decreased CETP activity and allele type (B1, B2)^9,10^. Allele B1 affects the size and function of the CETP protein and HDL-C level. In contrast, allele B2 is associated with lower-molecular-weight CETP and increased HDL-C. Moreover, increased HDL-C levels were associated with reduced CETP activity due to alcohol consumption^11^, and high alcohol consumption increased HDL-C levels in B2 carriers^12^.

Facial flushing in response to alcohol consumption, known as Asian flushing or the Asian glow, is a reaction specific to East Asian populations^13^. This response can estimate the activities of ALDH2 and ADH1B, which are the most efficient enzymes associated with aldehydes. Higher activity of ADH (conferred by Arg48) or lower activity of ALDH2 (conferred by Lys487) leads to accumulation of acetaldehyde following alcohol consumption. In particular, the *ALDH2* rs671 variant slows the degradation of acetaldehyde, and the accumulated acetaldehyde can cause serious inconvenience. In addition, *ALDH2* rs671 had different effects on HDL-C and total cholesterol according to genotype; lower HDL-C and higher total cholesterol levels were observed with the *ALDH2* rs671 genotype when compared to those observed in its absence^14^. Furthermore, carriers with the *ALDH2* rs671 GG genotype had substantially higher mean alcohol consumption and HDL-C levels than those with the other genotypes^15^. The GG genotype carriers in the Shandong province of China had a higher mean lipid level and total cholesterol disorder rate than those of the other genotype carriers^16^.

In this regard, ALDH2 variation should be considered as the main genetic proxy for the differences in cholesterol according to alcohol consumption. Moreover, it is important in alcohol research because of its direct relation to alcohol metabolism. However, few studies have addressed the influence of alcohol consumption on cholesterol according to CETP and ALDH2 variant type status. Accordingly, we aimed to investigate the association between alcohol consumption and cholesterol according to CETP and ALDH2 variants in the Korean population.

## Results

Data were obtained from the Health Examinees Study (HEXA) and Korean National Health and Nutrition Examination Survey (KNHANES). The characteristics of the study participants, according to sex, are presented in Table 1. Age, systolic/diastolic blood pressure, fasting glucose level, total cholesterol level, HDL-C level, TG level, aspartate transaminase (AST) level, alanine transaminase (ALT) level, and alcohol consumption rate differed by sex in both the HEXA and KNHANES study populations (*p* < 0.05). The frequency of *ALDH2* rs671 differed significantly by sex in the HEXA population, but not in the KNHANES population. The general characteristics of age, systolic/diastolic blood pressure, HDL-C level, TG level, and AST and ALT levels differed according to alcohol consumption in both study populations (Online Supplementary Table S1).

**Table 1:** General characteristics of Korean population.

	Total	Men	Women	*p*-value
**HEXA**				
Subject (n, %)	53,605(100.0)	18,482(34.5)	35,123(65.5)	
Age (years)	53.8 ± 8.0	55.2 ± 8.4	53.1 ± 7.7	< 0.0001
Systolic blood pressure (mmHg)	122.5 ± 14.8	125.6 ± 14.1	120.8 ± 14.9	< 0.0001
Diastolic blood pressure (mmHg)	75.8 ± 9.7	78.3 ± 9.5	74.5 ± 9.5	< 0.0001
Fasting glucose (mg/dL)	95.0 ± 19.6	99.1 ± 22.1	92.8 ± 17.8	< 0.0001
Total cholesterol (mg/dL)	197.4 ± 35.7	192.3 ± 34.9	200.1 ± 35.8	< 0.0001
HDL-cholesterol (mg/dL)	53.8 ± 13.1	49.2 ± 11.9	56.2 ± 13.1	< 0.0001
LDL-cholesterol (mg/dL)	119.3 ± 32.2	114.8 ± 31.5	121.7 ± 32.3	< 0.0001
Triglycerides (mg/dL)	125.1 ± 85.6	147.6 ± 101.7	113.3 ± 73.0	< 0.0001
AST (IU/L)	23.7 ± 23.2	25.4 ± 12.8	22.8 ± 27.1	< 0.0001
ALT (IU/L)	22.4 ± 22.8	26.5 ± 18.8	20.2 ± 24.3	< 0.0001
Alcohol consumption (Drinker, %)	23,751(44.3)	13,177(71.3)	10,574(30.1)	< 0.0001
*CETP* rs708272				
CC	20,523(38.3)	7,122(38.5)	13,401(38.2)	0.4276
CT	25,225(47.1)	8,698(47.1)	16,527(47.1)	
TT	7,857(14.7)	2,662(14.4)	5,195(14.8)	
*ALDH2* rs671				
GG	37,794(70.7)	12,904(69.9)	24,890(71.1)	0.0204
GA	14,443(27.0)	5,094(27.6)	9,349(26.7)	
AA	1,241(2.3)	541(2.4)	790(2.3)	
**KNHANES**				
Subject (n, %)	14,133(100.0)	6,893(48.8)	7,240(51.2)	
Age (years)	46.6 ± 16.5	47.0 ± 17.0	46.3 ± 16.0	< 0.0001
Systolic blood pressure (mmHg)	117.6 ± 16.2	120.4 ± 15.2	114.9 ± 16.7	< 0.0001
Diastolic blood pressure (mmHg)	75.4 ± 10.5	77.7 ± 10.4	73.3 ± 9.7	< 0.0001
Fasting glucose (mg/dL)	98.4 ± 21.7	100.8 ± 23.2	96.1 ± 19.9	< 0.0001
Total cholesterol (mg/dL)	188.0 ± 36.1	186.3 ± 35.4	189.6 ± 35.5	< 0.0001
HDL-cholesterol (mg/dL)	50.6 ± 12.2	47.5 ± 11.3	53.5 ± 12.4	< 0.0001
LDL-cholesterol (mg/dL)	117.5 ± 34.0	114.8 ± 34.0	120.9 ± 33.6	< 0.0001
Triglycerides (mg/dL)	135.5 ± 111.7	156.9 ± 131.6	115.0 ± 83.9	< 0.0001
AST (IU/L)	22.4 ± 13.1	24.3 ± 13.5	20.5 ± 12.2	< 0.0001
ALT (IU/L)	21.9 ± 19.0	25.9 ± 21.3	18.0 ± 15.6	< 0.0001
Alcohol consumption (Drinker, %)	12,544(88.8)	6,422(93.2)	6,122(84.6)	< 0.0001
*CETP* rs708272				
CC	5,481(38.8)	5,481(38.8)	2,844(39.3)	0.2496
CT	6,589(46.6)	3,263(47.3)	3,326(45.9)	
TT	2.063(14.6)	993(14.4)	1,070(14.8)	
*ALDH2* rs671				
GG	10,028(71.6)	4,852(70.5)	5,176(71.6)	0.3302
GA	3,737(26.5)	1,861(27.0)	1,876(26.0)	
AA	347(2.5)	171(2.5)	176(50.7)	

All data except alcohol consumption, *CETP* and *ALDH2* genotype are represented as means ± standard deviation. Student’s t-test and chi-square tests were used to determine differences between men and women.

*HDL* high-density lipoprotein, *AST* aspartate aminotransferase, *ALT* alanine aminotransferase.

The participants’ clinical characteristics are summarized according to *CETP* rs708272 [CC, CT, or TT] status in Table 2. The total cholesterol and HDL-C levels of *CETP* rs708272 risk T allele carriers were significantly higher than others in the HEXA and KNHANES populations (*p* < 0.05). In addition, the risk allele T carriers had a higher LDL-C levels in the KNHANES study (*p* < 0.05). When categorized by sex, LDL-C levels were significantly different between men in both the HEXA and KNHANES studies. (Online Supplementary Tables S2 and S3). Men carrying *CETP* rs708272 TT allele had higher LDL-C levels than those carrying the CT of the TT genotype. On the other hand, almost all general characteristics according to the *ALDH2* rs671 genotype, except for sex and total cholesterol, showed differences in both study populations (Online Supplementary Table S4).

**Table 2:** General characteristics of subjects according to *CETP* genotype in HEXA and KNHANES.

	*CETP*			*p* value
	CC	CT	TT	
**HEXA**				
Subject (n, %)	20,523(38.3)	25,225(47.1)	7,857(14.7)	
Age (years)	53.8 ± 8.0	53.8 ± 8.0	53.9 ± 8.0	0.8017
Sex (male)	7,122(34.7)	8,698(34.5)	2.662(33.9)	0.4276
Systolic blood pressure (mmHg)	122.5 ± 14.8	122.4 ± 14.8	122.5 ± 14.7	0.6488
Diastolic blood pressure (mmHg)	75.8 ± 9.7	75.8 ± 9.7	75.8 ± 9.8	0.6183
Fasting glucose (mg/dL)	95.0 ± 19.8	94.9 ± 19.7	94.9 ± 18.5	0.8042
Total cholesterol (mg/dL)	196.1 ± 35.5	197.7 ± 35.8	200.0 ± 35.9	< 0.0001
HDL-cholesterol (mg/dL)	52.2 ± 12.6	54.3 ± 13.2	56.4 ± 13.8	< 0.0001
LDL-cholesterol (mg/dL)	119.3 ± 32.0	119.3 ± 32.3	119.6 ± 32.1	0.6610
Triglycerides (mg/dL)	126.9 ± 86.5	124.2 ± 85.1	123.2 ± 84.4	0.0004
AST (IU/L)	23.9 ± 33.2	23.7 ± 13.8	23.6 ± 13.3	0.5443
ALT (IU/L)	22.6 ± 29.3	22.3 ± 17.2	22.3 ± 18.8	0.3703
Alcohol consumption (Drinker, %)	9,051(44.1)	11.218(44.8)	3,482(44.3)	0.2054
**KNHANES**				
Subject (n, %)	5,802(38.9)	6,949(46.5)	2,180(14.6)	
Age (years)	46.5 ± 16.9	46.7 ± 16.8	46.4 ± 16.6	0.6785
Sex (male)	2,783(48.0)	3,450(50.0)	1,048(48.1)	0.1310
Systolic blood pressure (mmHg)	117.5 ± 16.5	117.7 ± 16.3	117.6 ± 15.9	0.7698
Diastolic blood pressure (mmHg)	75.2 ± 10.6	75.3 ± 10.5	75.4 ± 10.6	0.6617
Fasting glucose (mg/dL)	98.5 ± 22.2	98.5 ± 22.0	98.8 ± 22.5	0.8674
Total cholesterol (mg/dL)	185.8 ± 36.1	188.6 ± 36.3	191.4 ± 35.9	< 0.0001
HDL-cholesterol (mg/dL)	49.3 ± 11.9	50.9 ± 12.3	52.7 ± 12.3	< 0.0001
LDL-cholesterol (mg/dL)	115.0 ± 33.7	119.2 ± 34.1	118.5 ± 33.8	< 0.0001
Triglycerides (mg/dL)	135.2 ± 114.1	134.9 ± 107.3	137.0 ± 115.8	0.7352
AST (IU/L)	22.7 ± 15.8	22.3 ± 11.6	22.3 ± 10.7	0.1381
ALT (IU/L)	22.2 ± 22.9	21.6 ± 16.8	22.0 ± 17.3	0.2100
Alcohol consumption (Drinker, %)	3,842(66.2)	4,654(67.0)	1,432(65.7)	0.4605

All data except alcohol consumption and sex are represented as means ± standard deviation. General linear models and chi-square tests were used to determine differences between *CETP* genotype.

*HDL* high-density lipoprotein, *AST* aspartate aminotransferase, *ALT* alanine aminotransferase.

Table 3 shows the total cholesterol and HDL-C levels according to alcohol consumption in carriers of *CETP* rs708272. HDL-C levels differed significantly between *CETP* rs708272 by alcohol consumption and sex in the HEXA and KNHANES study populations. In the HEXA population, *CETP* rs708272 was associated with a higher HDL-C level in drinkers than in non-drinkers of both sexes. However, HDL-C level did not differ with the association between the *CETP* rs708272 TT minor homozygous genotype and alcohol consumption in both sexes in the KNHANES population. In the HEXA population, total cholesterol varied significantly with alcohol consumption in men with *CETP* rs708272, whereas in women, there was no association between drinking and *CETP* rs708272. In addition, in the KNHANES population, total cholesterol was not associated with *CETP* rs708272 among non-drinkers and drinkers. In the HEXA population, total cholesterol differed significantly among men with *CETP* rs708272 depending on alcohol consumption. In contrast, the KNHANES population, there was no association between drinking status and *CETP* rs708272. In addition, *CETP* rs708272 was associated with higher total cholesterol levels in male drinkers than in non-drinkers in the HEXA population, whereas no association was found in the KNHANES population. In women, total cholesterol differed significantly among *CETP* rs708272 in non-drinkers in both the HEXA and KNHANES populations. Total cholesterol was inversely associated with alcohol consumption according to *CETP* rs708272 in the HEXA population. However, the only risk TT minor homozygous genotype of *CETP* rs708272 was inversely associated with higher total cholesterol in drinkers in the KNHANES population. Conversely, the association of cholesterol with alcohol consumption in the *CETP* rs708272 major homozygous genotype group disappeared.

**Table 3:** Association between alcohol consumption and cholesterol (HDL and total) according to *CETP* genotype.

	*CETP*			*p* value^1^
	CC	CT	TT	
**HEXA**				
**HDL-cholesterol**				
**Total**				
Non-drinker	52.2 ± 12.3	54.3 ± 12.8	56.4 ± 13.4	< 0.0001
Drinker	52.5 ± 13.0	54.8 ± 13.7	56.7 ± 14.1	< 0.0001
*p* value^2^	0.0468	0.0156	0.3931	
**Men**				
Non-drinker	44.9 ± 10.2	46.7 ± 11.0	48.4 ± 11.2	< 0.0001
Drinker	48.6 ± 11.4	51.1 ± 12.4	53.0 ± 12.7	< 0.0001
*p* value^2^	< 0.0001	< 0.0001	< 0.0001	
**Women**				
Non-drinker	53.4 ± 12.2	55.5 ± 12.6	57.6 ± 13.3	< 0.0001
Drinker	57.4 ± 13.2	59.4 ± 13.8	61.2 ± 14.5	< 0.0001
*p* value^2^	< 0.0001	< 0.0001	< 0.0001	
**Total cholesterol**				
**Total**				
Non-drinker	197.3 ± 35.7	199.0 ± 36.1	201.3 ± 35.9	< 0.0001
Drinker	195.3 ± 34.9	197.1 ± 35.2	198.8 ± 35.8	< 0.0001
*p* value^2^	< 0.0001	< 0.0001	0.0026	
**Men**				
Non-drinker	187.3 ± 35.0	190.1 ± 35.6	193.3 ± 34.5	0.0017
Drinker	192.1 ± 34.0	194.6 ± 34.4	197.4 ± 35.7	< 0.0001
*p* value^2^	< 0.0001	< 0.0001	0.0159	
**Women**				
Non-drinker	199.0 ± 35.5	200.4 ± 36.0	202.6 ± 35.9	< 0.0001
Drinker	199.3 ± 35.6	200.3 ± 36.0	200.6 ± 35.9	0.2872
*p* value^2^	0.7260	0.8584	0.0659	
**KNHANES**				
**HDL-cholesterol**				
**Total**				
Non-drinker	48.3 ± 11.2	50.2 ± 11.6	52.7 ± 11.7	< 0.0001
Drinker	48.0 ± 12.1	49.8 ± 12.4	51.3 ± 12.7	< 0.0001
*p* value^2^	0.5259	0.3307	0.0548	
**Men**				
Non-drinker	41.9 ± 8.8	45.0 ± 10.1	47.2 ± 9.3	0.0342
Drinker	45.5 ± 11.0	47.4 ± 11.7	49.6 ± 12.2	< 0.0001
*p* value^2^	0.0053	0.0366	0.3846	
**Women**				
Non-drinker	47.5 ± 10.2	50.0 ± 53.7	53.7 ± 10.1	< 0.0001
Drinker	51.4 ± 12.3	53.0 ± 12.3	53.6 ± 12.9	< 0.0001
*p* value^2^	< 0.0001	< 0.0001	0.9451	
**Total cholesterol**				
**Total**				
Non-drinker	189.4 ± 33.6	191.0 ± 33.9	198.7 ± 34.8	< 0.0001
Drinker	189.9 ± 34.4	192.5 ± 35.0	193.9 ± 33.7	0.0025
*p* value^2^	0.7471	0.2212	0.0227	
**Men**				
Non-drinker	184.4 ± 35.1	184.9 ± 30.0	187.6 ± 27.5	0.9300
Drinker	184.1 ± 32.4	186.1 ± 32.1	188.9 ± 27.9	0.0078
*p* value^2^	0.9541	0.7186	0.8351	
**Women**				
Non-drinker	189.7 ± 34.2	197.2 ± 36.9	207.3 ± 37.8	< 0.0001
Drinker	195.8 ± 34.9	197.6 ± 35.9	198.9 ± 37.4	0.1620
*p* value^2^	0.0043	0.8190	0.0205	

Data are expressed as means ± standard deviations.

^1^General linear models were used to assess differences in variables between *CETP* genotype.

^2^Student’s t-test was used to assess difference in a variable between alcohol consumption in each *CETP* genotype.

In the case of *ALDH2* rs671, HDL-C showed a significant difference according to the *ALDH2* rs671 genotype in non-drinkers and drinkers in the HEXA population. Nevertheless, only drinkers showed differences in the KNHANES population. Moreover, *ALDH2* rs671 [GA + AA] genotype was associated with lower HDL-C levels in drinkers than those in non-drinkers in the HEXA population. Total cholesterol in the HEXA and KNHANES populations differed according to the *ADLH2* rs671 genotype in drinkers (Online Supplementary Table S5).

The effects of *CETP* rs708272 and alcohol consumption on HDL-C according to *ALDH2* rs671 are shown in Table 4 and Online Supplementary Fig. S1. HDL-C differed significantly according to *CETP* rs708272 independent of *ALDH2* rs671 and alcohol consumption, but *CETP* rs708272 and HDL-C were slightly associated with the *ALDH2* rs671 [GA + AA] genotypes. In addition, those with the *ALDH2* rs671 [GA + AA] genotype exhibited trends toward lower HDL-C compared with those with the *ALDH2* rs671 GG major homozygous genotype. Still, overall, the HDL-C level was higher in men drinkers than in non-drinkers, regardless of the *ALDH2* rs671 genotype.

**Table 4:** Effects of *CETP* genotype and alcohol consumption on HDL-cholesterol according to *ALDH2* genotype.

	*ALDH2*(GG)		*ALDH2*(GA + AA)		*p* value^1^
	Non-drinker	Drinker	Non-drinker	Drinker	
**HEXA**					
**Total**					
CC	52.7 ± 12.5	53.0 ± 13.1	51.5 ± 12.0	50.1 ± 12.0	< 0.0001
CT	55.0 ± 12.8	55.2 ± 13.7	53.4 ± 12.7	52.4 ± 12.9	< 0.0001
TT	57.0 ± 13.3	57.0 ± 14.3	55.6 ± 13.6	54.9 ± 13.1	< 0.0001
*p* value^2^	< 0.0001	< 0.0001	< 0.0001	< 0.0001	
**Men**					
CC	45.6 ± 11.1	49.0 ± 11.5	44.6 ± 9.7	47.2 ± 10.6	< 0.0001
CT	47.9 ± 11.6	51.4 ± 12.5	46.0 ± 10.6	49.6 ± 11.5	< 0.0001
TT	49.6 ± 12.5	53.2 ± 12.8	47.9 ± 10.5	52.3 ± 12.5	< 0.0001
*p* value	< 0.0001	< 0.0001	< 0.0001	< 0.0001	
**Women**					
CC	53.3 ± 12.5	57.5 ± 13.3	53.5 ± 11.8	56.7 ± 12.5	< 0.0001
CT	55.6 ± 12.7	59.5 ± 13.8	55.4 ± 12.4	58.9 ± 13.6	< 0.0001
TT	57.6 ± 13.2	61.3 ± 14.7	57.8 ± 13.6	60.9 ± 12.7	< 0.0001
*p* value	< 0.0001	< 0.0001	< 0.0001	0.0006	
**KNHANES**					
**Total**					
CC	46.6 ± 9.9	49.0 ± 12.3	46.3 ± 10.5	46.4 ± 11.1	< 0.0001
CT	49.3 ± 11.1	50.6 ± 12.4	48.8 ± 11.1	48.4 ± 11.9	< 0.0001
TT	53.1 ± 11.2	52.1 ± 12.7	52.7 ± 9.4	50.0 ± 12.7	0.0566
*p* value	< 0.0001	< 0.0001	< 0.0001	< 0.0001	
**Men**					
CC	39.9 ± 11.1	46.4 ± 11.4	42.2 ± 8.4	43.5 ± 9.7	< 0.0001
CT	46.7 ± 11.1	48.4 ± 11.9	44.3 ± 9.8	44.9 ± 10.7	< 0.0001
TT	50.5 ± 5.4	50.5 ± 12.4	46.4 ± 10.0	47.3 ± 11.4	0.0239
*p* value	0.1561	< 0.0001	0.1883	0.0002	
**Women**					
CC	47.1 ± 9.7	51.8 ± 12.5	48.0 ± 10.7	50.3 ± 11.6	< 0.0001
CT	49.7 ± 11.1	53.0 ± 12.4	50.4 ± 11.2	53.2 ± 11.8	0.0002
TT	53.2 ± 11.4	53.7 ± 12.7	54.2 ± 8.7	53.3 ± 13.3	0.9532
*p* value	0.0003	0.0090	0.0003	0.0018	

Data are expressed as means ± standard deviations.

^1^General linear models by *CETP* genotypes in each group, and differences between alcohol consumption and *ALDH2* genotype.

^2^General linear models were used to assess *CETP* genotype differences in variables between alcohol consumption and HDL-cholesterol by *ALDH2* genotype.

Table 5 shows the association of total cholesterol with alcohol consumption and *ALDH2* rs671 according to *CETP* rs708272. In men, total cholesterol was associated with alcohol consumption and *ADLH2* rs671 when *CETP* rs708272 CT heterozygous and TT minor homozygous genotypes were present in both HEXA and KNHANES populations. Additionally, drinkers with *ALDH2* rs671 exhibited the highest total cholesterol level with the *CETP* rs708272 TT risk minor homozygous genotype in both the HEXA and KNHANES populations. On the other hand, as a result of analyzing the association between the *CETP* rs708272 genotype and alcohol consumption according to *ALDH2* variation in women, the total cholesterol level was high in the presence of the *CETP* rs708272 risk T allele, but there was no significant difference, unlike in men. In particular, male drinkers with the *CETP* rs708272 TT risk minor homozygous genotype had the highest total cholesterol level when they had the *ALDH2* rs671 [GA + AA] genotype.

**Table 5:** Effects of *CETP* genotype and alcohol consumption on total cholesterol according to *ALDH2* genotype.

	*ALDH2*(GG)		*ALDH2*(GA + AA)		*p* value^1^
	Non-drinker	Drinker	Non-drinker	Drinker	
**HEXA**					
**Total**					
CC	197.3 ± 35.5	195.7 ± 35.0	197.2 ± 35.9	193.2 ± 34.4	< 0.0001
CT	199.3 ± 36.4	197.6 ± 35.2	201.7 ± 35.8	200.3 ± 34.2	< 0.0001
TT	201.1 ± 35.9	198.5 ± 36.2	201.7 ± 35.8	200.3 ± 34.2	0.0121
*p* value^2)^	< 0.0001	0.0001	< 0.0001	0.0002	
**Men**					
CC	185.0 ± 34.0	192.7 ± 34.4	188.5 ± 35.5	190.1 ± 32.4	< 0.0001
CT	188.1 ± 35.2	194.8 ± 34.5	191.2 ± 35.7	193.9 ± 34.2	< 0.0001
TT	189.2 ± 31.6	196.5 ± 36.1	195.1 ± 35.6	200.9 ± 33.8	0.0031
*p* value^2)^	0.2019	0.0004	0.0069	< 0.0001	
**Women**					
CC	198.5 ± 35.4	199.1 ± 35.4	199.8 ± 35.6	200.0 ± 37.7	0.3288
CT	200.3 ± 36.3	200.7 ± 35.8	200.6 ± 35.3	196.8 ± 37.0	0.1015
TT	202.0 ± 36.0	200.7 ± 36.1	203.5 ± 35.7	199.0 ± 35.2	0.1479
*p* value^2^	0.0002	0.1065	0.0057	0.3711	
**KNHANES**					
**Total**					
CC	189.0 ± 35.2	189.5 ± 34.4	188.6 ± 33.8	190.8 ± 33.6	0.7448
CT	195.8 ± 36.4	191.1 ± 34.0	193.9 ± 35.6	193.2 ± 35.8	0.0959
TT	199.0 ± 35.2	192.8 ± 34.2	210.1 ± 38.4	197.3 ± 31.1	< 0.0001
*p* value^2^	0.0738	0.0477	< 0.0001	0.0209	
**Men**					
CC	173.3 ± 29.3	182.8 ± 31.4	185.9 ± 35.8	187.2 ± 34.3	0.0641
CT	181.1 ± 32.7	184.7 ± 30.5	186.2 ± 29.2	189.4 ± 35.2	0.0304
TT	185.0 ± 38.4	186.4 ± 28.7	188.3 ± 25.5	195.3 ± 24.4	0.0125
*p* value^2^	0.7811	0.0983	0.9691	0.0437	
**Women**					
CC	189.9 ± 35.4	196.0 ± 35.8	189.6 ± 33.1	195.6 ± 32.1	0.0400
CT	197.8 ± 36.5	197.4 ± 36.0	196.5 ± 37.3	198.4 ± 35.9	0.9386
TT	199.8 ± 35.2	198.8 ± 37.7	214.6 ± 39.2	199.5 ± 36.8	0.0146
*p* value^2^	0.0595	0.3612	< 0.0001	0.4070	

Data are expressed as means ± standard deviations.

^1^General linear models by *CETP* genotypes in each group, and differences between alcohol consumption and *ALDH2* genotype.

^2^General linear models were used to assess *CETP* genotype differences in variables between alcohol consumption and total cholesterol by *ALDH2* genotype.

## Discussion

Several studies have reported associations between *CETP* rs708272, alcohol consumption, and cholesterol levels. In addition, *ALDH2* rs671, which is closely related to alcohol consumption in East Asians, is well known to be associated with HDL-c according to alcohol consumption^17^, but studies involving CETP variants have not been reported. We investigated the association between alcohol consumption and cholesterol levels according to CETP and ALDH2 variants and their combinations in two large-scale Korean populations.

Previous studies have shown that *CETP* rs708272 increases HDL-C levels. In a meta-analysis of 98 published studies^18^, *CETP* rs708272 was associated with higher mean HDL-C levels. In that study, HDL-C level was higher in the *CETP* rs708272 risk allele (TT and CT genotype) carriers than in the CC major homozygous genotype carriers. In 2018, Cai *et. al.* reported that the HDL level of participants with *CETP* rs708272 TT genotype was higher than those of CC genotype, and the HDL level of male participants with only T allele was significantly higher^19^. Also, some studies have shown a gene × alcohol consumption interaction for CETP. Based on the limited evidence, alcohol consumption appears to be associated with lower CETP activity^20^. In the association between alcohol consumption and HDL-C, changes in other metabolic pathways including an increased transport rate of apolipoproteins, reduced hepatic lipase activity, and so on may be likely^21–25^. However, our study found no interaction between *CETP* rs708272 and alcohol consumption in determining HDL-C. This is in line with previous studies carried out in a Mediterranean population^26^, healthy men^27^, and insulin-dependent men^28^. The metabolic mechanisms underlying an increase in HDL-C following the interaction between CETP and alcohol consumption are poorly understood, and more research is needed.

In this study, we observed that increased HDL-C was associated with alcohol consumption independent of *ALDH2* rs671; this result is similar to that of a previous meta-analysis, which revealed an association between alcohol consumption and HDL-C. However, Wakabayashi et al. reported that the effect of alcohol consumption on HDL-C was different in drinkers with an alcohol flushing response^29^. The beneficial effects of an association between alcohol consumption and HDL-C according to *ALDH2* rs671 remains controversial because several other studies have shown that HDL-C is influenced by alcohol consumption differently in drinkers with an alcohol flushing response. According to some data, the *ALDH2* rs671 A allele carriers, GA, and AA genotypes have a 2.6-fold higher risk of hypo-HDL-cholesterolemia than the GG homozygous genotype^30^. These conflicting findings may be due to the small size of the study population. Further studies using larger populations are needed to clarify whether ALDH2 affects the relationship between alcohol consumption and HDL-C.

The effects of alcohol consumption on total cholesterol may differ according to the CETP or ALDH2 status. Previous studies have shown that alcohol consumption in subjects with the *ALDH2* rs671 risk A allele was associated with increased total cholesterol compared to alcohol consumption with the *ALDH2* rs671 GG homozygous genotype^31^. Similar to previous studies, our study found that drinkers with the *ALDH2* [GA + AA] genotype had higher total cholesterol levels than non-drinkers with the *ALDH2* GG homozygous genotype. CETP variants also showed an association with total cholesterol increase^32^, but no significant difference was found in total cholesterol levels according to alcohol consumption in *CETP* rs708272^33^. The total cholesterol levels in our study were the highest in both men and women with *CETP* rs708272 risk TT genotype. Still, there was no difference according to *ALDH2* rs671 genotype, while the presence of *ALDH2* rs671 risk alleles in the drinking men group with *CETP* rs708272 risk TT genotype was associated with changes in total cholesterol levels.

This was an observational study of the association between alcohol consumption and cholesterol according to CETP and ALDH2 variants. Residual confounding is a critical issue that should be considered in observational studies. We replicated two Korean population cohorts (HEXA and KNHANES) to address residual confounding. In the present study, we revealed differences in HDL-cholesterol according to the presence of *CETP* rs708272, independent of *ALDH2* rs671 and alcohol consumption, and found that the presence of *CETP* rs708272 risk allele in men was associated with an increase in HDL cholesterol following alcohol intake. Furthermore, total cholesterol levels were associated with alcohol consumption and *ALDH2* rs671 in men with *CETP* rs708272 CT and TT genotypes with strong genetic effects. This study provides information about genetic variants of CETP and ALDH2 in the Korean population. It clarifies the probable mechanism underlying the differential association between alcohol consumption and cholesterol (total cholesterol and HDL-C) according to CETP and ALDH2 variants.

Overall, our work would be helpful in clarifying the clinical implications of improving the translational value for preventive management of public health policies, and further realizing personalized or precision medicines that can prevent individual diseases or realize early detection, treatment decisions, and prognosis. It can also be used as a predictor of population assessment and vulnerable groups, while providing evidence for health policy development and health insurance applications.

## Methods

### Study population

Data were obtained from two studies conducted by the Korea Disease Control and Prevention Agency (KDCA) as part of the Korean Genome and Epidemiology Study (KoGES) and the KNHANES. Written informed consent was obtained from all participants, and the research project was approved by KDCA. The study protocol was approved by the Institutional Review Board of the Korean National Institute of Health (2019-03-01-PE-A). HEXA and KNHANES have been previously described in detail^34^. All study protocols were carried out by the approved guidelines.

The HEXA cohort subjects (*n* = 53,754) were invited to health examination centers in eight regions (metropolitan areas or major cities) and enrolled in 38 health examination centers and hospitals in Korea between 2004 and 2013. Individuals with missing data for the *CETP* rs708272 genotype (*n* = 149) or alcohol consumption (*n* = 2256) were excluded. A total of 51,349 participants were included in this cross-sectional study.

KHNANES is a nationally representative survey of the health and nutritional status of the Korean population. We used data from a population of 15,000 people aged 20–60 years collected during the 2009–2015 period. Individuals with missing data for alcohol consumption (*n* = 401), cholesterol (HDL and total cholesterol, *n* = 153), and those under 40 years of age (*n* = 5288) were excluded. In total, 9158 participants were included in this cross-sectional study.

### Anthropometric and biochemical analyses

In HEXA, age and smoking status information was obtained using interview-based questionnaires. Body mass index (BMI) was calculated as weight divided by height squared. The lipid profile (total cholesterol, TGs, and HDL-C) and fasting glucose, ALT, and AST levels were measured using a Hitachi 747 chemistry analyzer (Hitachi, Ltd., Tokyo, Japan). Systolic/diastolic blood pressure was measured twice using a standard mercury sphygmomanometer, and the results were averaged.

In the KNHANES, data were collected by various means, including interview-based and self-reported questionnaires, physical examinations, and assessments of nutritional status. The calculation of BMI and blood pressure (systolic and diastolic) was the same as that for HEXA. The lipid profile (total cholesterol, TGs, and HDL-C) and plasma glucose, ALT, and AST levels were measured using a Hitachi Automatic Analyzer 7600 (Hitachi, Tokyo, Japan).

Alcohol consumption data were collected using an interview-based questionnaire in HEXA and KNHNES. Subjects were asked whether they had ever consumed at least one alcoholic drink every month, and if they had, whether they were former-drinker or current drinkers^35^.

### Genotyping

HEXA cohort subjects were genotyped using a Korean chip designed by the Center for Genome Science at the Korean National Institute of Health. The chip was provided and its use was approved by the National Biobank of Korea, the Centers for Disease Control and Prevention, Republic of Korea (No. 2019-022). The genotyping protocol and quality control process has been described in detail previously^36^.

We preliminarily analyzed the association between cholesterol and genes using 128 SNPs related to alcohol metabolism which were selected from the KNHANES by TaqMan genotyping assay as previously described^37^. Only *CETP* rs708272 and *ALDH2* rs671 were related to cholesterol in two sets of Korean population data, which led to the use of this study.

### Statistical analysis

Statistical analyses were performed using SAS software package (ver. 9.4; SAS Institute Inc., Cary, NC, USA). Data are presented as the mean ± standard deviation or number (%). Logarithmic transformation was applied to variables with a non-Gaussian distribution. Student’s t-tests were conducted to compare clinical characteristics according to sex, and chi-square tests were used for categorical variables (ALDH2, CETP, and alcohol consumption). Analysis of variance was conducted to compare alcohol consumption according to *CETP* and *ALDH2* genotypes. Multivariable linear regression models were used to assess differences in variables between *CETP* and *ALDH2* genotypes and the combined effects of *CETP* and *ALDH2* on the association between alcohol consumption and cholesterol, adjusted for age, BMI, and smoking. Statistical tests were two sided, and values of *p* < 0.05 were considered indicative of statistical significance.

## Supplementary Information

Supplementary Information.
