# Supplementary material for: The effect of the association between CETP variant type and alcohol consumption on cholesterol level differs according to the ALDH2 variant type
Source: Sci Rep. 2022 Sep 6;12:15129. doi: 10.1038/s41598-022-19171-y (PMC9448738; doi:10.1038/s41598-022-19171-y)
Supplement: Supplementary file 1 — Supplementary Information. [file 41598_2022_19171_MOESM1_ESM.docx]

Title:

**The effect of the association between CETP variant type and alcohol consumption on cholesterol level differs according to the ALDH2 variant type**

Min-Gyu Yoo ^1,†^, Ji Ho Yun^1,†^, Hye-Ja Lee^1,*^

^1^Division of Endocrine and Kidney Disease Research, Department of Chronic Disease Convergence Research, Korea National Institute of Health, Korea Disease Control and Prevention Agency, 187 Osongsaengmyeong 2-ro, Osong-eup, Cheongju-si, Chungcheongbuk-do, 28159, Republic of Korea

**^†^These authors contributed equally as co-first authors**

**^*^Correspondence to:**

Hye-Ja Lee, PhD

Phone: +82-43-719-8692, Fax: +82-43-719-8602, Email: [hyejalee@yahoo.co.kr](mailto:hyejalee@yahoo.co.kr)

Supplementary table 1. General characteristics according to alcohol consumption

|  | **Non-drinker** | **Drinker** | ***p-value*** |
| --- | --- | --- | --- |
| **HEXA** |  |  |  |
| Subject (n, %) | 27.598(53.8) | 23,751(46.3) |  |
| Age (years) | 54.6±7.8 | 52.7±8.1 | <0.0001 |
| Systolic blood pressure (mmHg) | 121.5±14.9 | 123.4±14.6 | <0.0001 |
| Diastolic blood pressure (mmHg) | 74.8±9.6 | 76.8±9.8 | <0.0001 |
| Fasting glucose (mg/dL) | 93.7±18.8 | 96.2±20.0 | <0.0001 |
| Total cholesterol (mg/dL) | 198.7±35.9 | 196.7±35.2 | <0.0001 |
| HDL-cholesterol (mg/dL) | 53.8±12.8 | 54.2±13.6 | 0.0013 |
| LDL-cholesterol (mg/dL) | 121.7±32.4 | 117.0±31.7 | <0.0001 |
| Triglycerides (mg/dL) | 118.4±74.5 | 132.6±97.0 | <0.0001 |
| AST (IU/L) | 23.3±29.7 | 24.1±13.1 | <0.0001 |
| ALT (IU/L) | 21.4±25.6 | 23.2±18.7 | <0.0001 |
| **KNHANES** |  |  |  |
| Subject (n, %) | 1,589(11.2) | 12,544(88.8) |  |
| Age (years) | 61.7±9.7 | 55.9±10.2 | <0.0001 |
| Systolic blood pressure (mmHg) | 123.9±17.7 | 121.4±16.7 | <0.0001 |
| Diastolic blood pressure (mmHg) | 75.0±10.1 | 77.2±10.3 | <0.0001 |
| Fasting glucose (mg/dL) | 102.1±23.4 | 102.4±16.7 | 0.6379 |
| Total cholesterol (mg/dL) | 193.9±35.9 | 191.3±34.2 | 0.0212 |
| HDL-cholesterol (mg/dL) | 48.6±10.8 | 49.6±12.3 | 0.0124 |
| LDL-cholesterol (mg/dL) | 122.3±33.6 | 117.2±34.0 | <0.0001 |
| Triglycerides (mg/dL) | 135.8±75.1 | 148.5±118.1 | 0.0007 |
| AST (IU/L) | 22.2±8.6 | 23.9±14.5 | 0.0003 |
| ALT (IU/L) | 20.4±12.0 | 22.9±18.8 | <0.0001 |

Data are expressed as means ± standard deviation. Student t-tests were used to determine differences between men and women. HDL, high-density lipoprotein; AST, aspartate aminotransferase; ALT, alanine aminotransferase.

Supplementary table 2. General characteristics of subjcts according to *CETP* genotype in men

|  | ***CETP*** | | | ***p* value** |
| --- | --- | --- | --- | --- |
|  | **CC** | **CT** | **TT** |  |
| **HEXA** |  |  |  |  |
| Subject (n, %) | 7,122(38.5) | 8,698(47.1) | 2,662(14.4) |  |
| Age (years) | 55.2±8.4 | 55.2±8.4 | 55.2±8.3 | 0.8866 |
| Systolic blood pressure (mmHg) | 125.7±14.1 | 125.5±14.0 | 125.7±14.2 | 0.6057 |
| Diastolic blood pressure (mmHg) | 78.4±9.5 | 78.3±9.5 | 78.3±9.8 | 0.4550 |
| Fasting glucose (mg/dL) | 99.1±22.7 | 99.1±22.1 | 98.9±20.0 | 0.8602 |
| Total cholesterol (mg/dL) | 190.4±34.5 | 192.7±35.0 | 196.1±35.3 | <0.0001 |
| HDL-cholesterol (mg/dL) | 47.5±11.2 | 49.8±12.1 | 51.9±12.6 | <0.0001 |
| LDL-cholesterol (mg/dL) | 114.2±31.4 | 114.8±31.4 | 116.2±31.9 | 0.0180 |
| Triglycerides (mg/dL) | 149.3±101.2 | 146.4±25.4 | 146.8±105.3 | 0.1880 |
| AST (IU/L) | 25.6±13.4 | 25.4±12.7 | 25.3±11.3 | 0.5792 |
| ALT (IU/L) | 26.7±21.7 | 26.3±16.4 | 26.6±17.3. | 0.2946 |
| **KNHANES** |  |  |  |  |
| Subject (n, %) | 2,637(38.3) | 3,263(47.3) | 933(14.4) |  |
| Age (years) | 47.2±17.2 | 47.1±17.1 | 45.9±16.6 | 0.1134 |
| Systolic blood pressure (mmHg) | 120.5±77.6 | 120.4±15.2 | 119.8±14.3 | 0.4587 |
| Diastolic blood pressure (mmHg) | 77.6±10.8 | 77.6±10.7 | 78.0±10.8 | 0.5586 |
| Fasting glucose (mg/dL) | 101.0±23.9 | 100.8±22.8 | 100.0±22.4 | 0.5362 |
| Total cholesterol (mg/dL) | 184.1±36.2 | 187.2±35.9 | 189.6±35.5 | <0.0001 |
| HDL-cholesterol (mg/dL) | 46.1±10.9 | 47.8±11.3 | 49.8±11.7 | <0.0001 |
| LDL-cholesterol (mg/dL) | 111.9±34.4 | 116.4±33.9 | 117.0±33.0 | 0.0182 |
| Triglycerides (mg/dL) | 157.1±130.0 | 155.9±127.7 | 160.1±147.2 | 0.6739 |
| AST (IU/L) | 24.6±15.1 | 24.1±13.1 | 24.2±12.0 | 0.3469 |
| ALT (IU/L) | 26.2±24.4 | 25.6±19.2 | 26.0±18.9 | 0.5130 |

Data are expressed as means ± standard deviation. General linear models were used to assess differences in variables between *CETP* genotype. HDL, high-density lipoprotein; AST, aspartate aminotransferase; ALT, alanine aminotransferase.

Supplementary table 3. General characteristics of subjcts according to CETP genotype in women

|  | **CETP** | | | ***p* value** |
| --- | --- | --- | --- | --- |
|  | **CC** | **CT** | **TT** |  |
| **HEXA** |  |  |  |  |
| Subject (n, %) |  |  |  |  |
| Age (years) | 53.1±7.7 | 53.0±7.7 | 53.2±7.7 | 0.4073 |
| Systolic blood pressure (mmHg) | 120.8±14.9 | 120.8±14.9 | 120.9±14.7 | 0.8329 |
| Diastolic blood pressure (mmHg) | 74.5±9.5 | 74.4±9.5 | 74.5±9.6 | 0.9316 |
| Fasting glucose (mg/dL) | 92.9±17.7 | 92.7±17.9 | 92.9±17.4 | 0.6567 |
| Total cholesterol (mg/dL) | 199.1±35.6 | 200.4±35.9 | 201.9±36.0 | <0.0001 |
| HDL-cholesterol (mg/dL) | 54.6±12.7 | 56.7±13.1 | 58.7±13.7 | <0.0001 |
| LDL-cholesterol (mg/dL) | 121.9±32.0 | 121.6±32.5 | 121.3±32.1 | 0.4664 |
| Triglycerides (mg/dL) | 115.0±75.0 | 112.5±72.7 | 111.1±68.2 | 0.0010 |
| AST (IU/L) | 23.0±39.9 | 22.8±14.3 | 22.7±14.1 | 0.7516 |
| ALT (IU/L) | 20.3±32.4 | 20.1±17.2 | 20.1±19.1 | 0.7331 |
| **KNHANES** |  |  |  |  |
| Subject (n, %) | 2,844(39.3) | 3,326(45.9) | 1,070(14.8) |  |
| Age (years) | 45.9±16.0 | 46.5±15.9 | 46.9±16.0 | 0.1136 |
| Systolic blood pressure (mmHg) | 114.6±16.5 | 115.0±16.8 | 115.4±16.9 | 0.3747 |
| Diastolic blood pressure (mmHg) | 73.3±9.7 | 73.2±9.7 | 73.3±9.7 | 0.3942 |
| Fasting glucose (mg/dL) | 95.6±19.0 | 96.2±20.5 | 96.9±19.9 | 0.1968 |
| Total cholesterol (mg/dL) | 187.4±35.5 | 190.3±36.4 | 193.7±36.5 | <0.0001 |
| HDL-cholesterol (mg/dL) | 52.3±12.0 | 54.0±12.5 | 55.4±12.3 | <0.0001 |
| LDL-cholesterol (mg/dL) | 118.8±32.5 | 123.0±34.1 | 120.4±34.8 | 0.0891 |
| Triglycerides (mg/dL) | 114.8±92.2 | 115.0±79.8 | 115.8±72.6 | 0.9414 |
| AST (IU/L) | 20.6±15.4 | 20.3±9.7 | 20.5±9.1 | 0.6017 |
| ALT (IU/L) | 18.2±18.3 | 17.8±13.0 | 18.4±14.8 | 0.4036 |

Data are expressed as means ± standard deviation. General linear models were used to assess differences in variables between *CETP* genotype. HDL, high-density lipoprotein; AST, aspartate aminotransferase; ALT, alanine aminotransferase.

Supplementary table 4. General characteristics of subjcts accrding to *ALDH2* genotype in HEXA and KNHANES.

|  | ***ALDH2*** | | | ***p* value** |
| --- | --- | --- | --- | --- |
|  | **GG** | **GA** | **AA** |  |
| **HEXA** |  |  |  |  |
| Subject (n, %) | 37,794(70.7) | 14,443(27.0) | 1,241(2.3) |  |
| Age (years) | 53.7±8.0 | 54.1±8.1 | 54.1±8.1 | <0.0001 |
| Systolic blood pressure (mmHg) | 122.8±14.9 | 121.6±14.4 | 122.2±14.5 | <0.0001 |
| Diastolic blood pressure (mmHg) | 76.0±9.8 | 75.2±9.5 | 75.3±9.5 | <0.0001 |
| Fasting glucose (mg/dL) | 95.4±20.1 | 93.8±18.3 | 93.7±16.0 | <0.0001 |
| Total cholesterol (mg/dL) | 197.4±35.7 | 197.4±35.5 | 197.6±36.9 | 0.9734 |
| HDL-cholesterol (mg/dL) | 54.3±13.3 | 52.7±12.7 | 51.7±12.3 | <0.0001 |
| LDL-cholesterol (mg/dL) | 118.7±32.2 | 120.7±31.8 | 121.4±33.4 | <0.0001 |
| Triglycerides (mg/dL) | 126.2±88.6 | 122.3±77.8 | 124.6±76.5 | <0.0001 |
| AST (IU/L) | 24.0±26.8 | 23.1±10.9 | 22.9±8.8 | <0.0001 |
| ALT (IU/L) | 22.6±24.9 | 21.7±17.2 | 221. ±13.1 | <0.0001 |
| **KNHANES** |  |  |  |  |
| Subject (n, %) | 10,028(71.1) | 3,737(26.5) | 347(2.5) |  |
| Age (years) | 46.5±16.4 | 47.1±16.8 | 45.8±16.0 | 0.0862 |
| Systolic blood pressure (mmHg) | 118.0±16.3 | 116.5±16.0 | 115.2±15.4 | <0.0001 |
| Diastolic blood pressure (mmHg) | 75.7±10.6 | 74.8±10.0 | 74.0±9.7 | <0.0001 |
| Fasting glucose (mg/dL) | 98.9±22.3 | 97.1±19.9 | 96.8±19.9 | <0.0001 |
| Total cholesterol (mg/dL) | 188.3±36.5 | 187.1±35.1 | 190.4±35.0 | 0.0893 |
| HDL-cholesterol (mg/dL) | 51.2±12.3 | 49.0±11.7 | 49.3±12.4 | <0.0001 |
| LDL-cholesterol (mg/dL) | 115.8±33.7 | 121.8±34.2 | 124.3±33.8 | 0.0085 |
| Triglycerides (mg/dL) | 139.4±120.4 | 125.8±86.8 | 126.1±81.7 | <0.0001 |
| AST (IU/L) | 22.8±14.3 | 21.3±9.5 | 20.9±8.1 | <0.0001 |
| ALT (IU/L) | 22.3±20.0 | 20.8±16.3 | 21.8±15.2 | 0.0005 |

Data are expressed as means ± standard deviation. General linear models were used to assess differences in variables between *ALDH2* genotype. HDL, high-density lipoprotein; AST, aspartate aminotransferase; ALT, alanine aminotransferase.

Supplementary table 5. Association between alcohol consumption and cholesterol (HDL and total) according to *ALDH2* genotype.

|  | ***ALDH2*** | |  |
| --- | --- | --- | --- |
|  | **GG** | **GA+AA** | ***p* value^1)^** |
| **HEXA** |  |  |  |
| HDL-cholesterol |  |  |  |
| Total |  |  |  |
| Non-drinker | 54.4±12.9 | 53.0±12.6 | <0.0001 |
| Drinker | 54.6±13.7 | 51.9±12.7 | <0.0001 |
| *p* value^2)^ | 0.0976 | <0.0001 |  |
| Men |  |  |  |
| Non-drinker | 47.2±11.6 | 45.7±10.3 | <0.0001 |
| Drinker | 50.8±12.3 | 49.1±11.5 | <0.0001 |
| *p* value^2)^ | <0.0001 | <0.0001 |  |
| Women |  |  |  |
| Non-drinker | 55.0±12.8 | 55.0±12.5 | 0.9482 |
| Drinker | 59.0±13.8 | 58.3±13.1 | 0.1283 |
| *p* value^2)^ | <0.0001 | <0.0001 |  |
| Total cholesterol |  |  |  |
| Total |  |  |  |
| Non-drinker | 198.8±36.0 | 198.5±79.0 | 0.5042 |
| Drinker | 197.0±35.3 | 195.0±34.8 | 0.0012 |
| *p* value^2)^ | <0.0001 | <0.0001 |  |
| Men |  |  |  |
| Non-drinker | 187.0±34.3 | 190.7±35.7 | 0.0020 |
| Drinker | 194.2±34.7 | 193.5±33.6 | 0.3251 |
| *p* value^2)^ | <0.0001 | 0.0037 |  |
| Women |  |  |  |
| Non-drinker | 199.9±36.0 | 200.8±35.5 | 0.0623 |
| Drinker | 200.1±35.7 | 198.4±37.0 | 0.1118 |
| *p* value^2)^ | 0.5847 | 0.0302 |  |
| **KNHANES** |  |  |  |
| HDL-cholesterol |  |  |  |
| Total |  |  |  |
| Non-drinker | 48.9±10.9 | 48.4±10.8 | 0.4613 |
| Drinker | 50.2±12.4 | 47.8±11.8 | <0.0001 |
| *p* value^2)^ | 0.2465 | 0.3095 |  |
| Men |  |  |  |
| Non-drinker | 45.4±11.0 | 43.7±9.3 | 0.3108 |
| Drinker | 47.9±11.9 | 44.7±10.5 | 0.2481 |
| *p* value^2)^ | 0.1811 | 0.2506 |  |
| Women |  |  |  |
| Non-drinker | 49.2±10.8 | 50.1±10.8 | <0.0001 |
| Drinker | 52.6±12.5 | 52.1±12.0 | 0.2465 |
| *p* value^2)^ | <0.0001 | 0.0034 |  |
| Total cholesterol |  |  |  |
| Total |  |  |  |
| Non-drinker | 193.7±35.9 | 194.1±36.0 | 0.8824 |
| Drinker | 190.7±34.2 | 192.8±34.4 | 0.0195 |
| *p* value^2)^ | 0.0034 | 0.4422 |  |
| Men |  |  |  |
| Non-drinker | 179.7±31.8 | 186.3±31.7 | 0.2519 |
| Drinker | 184.2±30.6 | 189.2±33.8 | <0.0001 |
| *p* value^2)^ | 0.3643 | 0.3144 |  |
| Women |  |  |  |
| Non-drinker | 195.0±36.0 | 196.7±37.0 | 0.4932 |
| Drinker | 197.0±36.1 | 197.4±34.6 | 0.7790 |
| *p* value^2)^ | 0.2849 | 0.7407 |  |

Date are expressed as means ± standard deviations unless otherwise indicated. ^1)^ Student t-test were used to assess differences in variables between *ALDH2* genotype . ^2)^ Student t-test was used to assess difference in a variable between alcohol consumption in each *ALDH2* genotype.


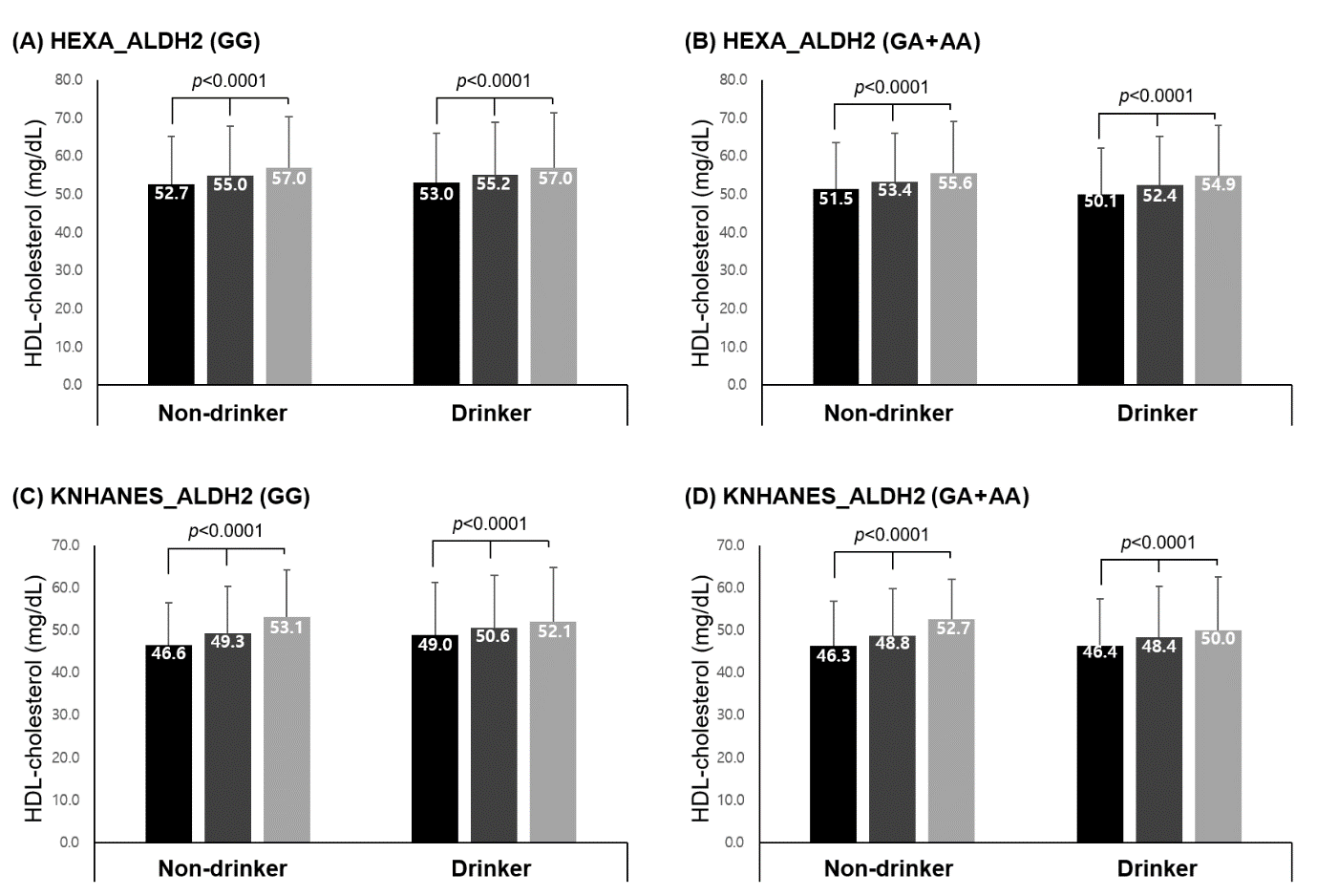


**Supplementary figure 1.** **Effect of *CETP* genotype and alcohol consumption on HDL-cholesterol according to *ALDH2*.** HDL-C level of *ALDH2* [GG] genotype (A and C) and *ALDH2* [GA+AA] genotype (B and D) according to alcohol consumption in HEXA and KNHANES. P-values were determined using general linear models for HDL-cholesterol according to *CETP* rs708272 (CC, black; CT, dark gray; TT; gray) and alcohol consumption.
